# Frequency of inter-specialty consensus decisions and adherence to advice following discussion at a weekly neurovascular multidisciplinary meeting

**DOI:** 10.1007/s11845-023-03319-4

**Published:** 2023-04-21

**Authors:** Chika Offiah, Sean Tierney, Bridget Egan, Ronán D. Collins, Daniel J. Ryan, Allan J. McCarthy, Deirdre R. Smith, James Mahon, Emily Boyle, Holly Delaney, Rory O.’Donohoe, Alison Hurley, Richard A. Walsh, Sinead M. Murphy, Petya Bogdanova-Mihaylova, Sean O.’Dowd, Mark J. Kelly, Taha Omer, Tara Coughlan, Desmond O’Neill, Mary Martin, Stephen J. X. Murphy, Dominick J. H. McCabe

**Affiliations:** 1 Dept. of Neurology, Tallaght University Hospital (TUH) / The Adelaide and Meath Hospital, Dublin, incorporating the National Children’s Hospital (AMNCH), Dublin, Ireland; 2 Stroke Service, Tallaght University Hospital (TUH) / The Adelaide and Meath Hospital, Dublin, incorporating the National Children’s Hospital (AMNCH), Dublin, Ireland; 3 Dept. of Vascular Surgery, Tallaght University Hospital (TUH) / The Adelaide and Meath Hospital, Dublin, incorporating the National Children’s Hospital (AMNCH), Dublin, Ireland; 4 Dept. of Age-Related Health Care, Tallaght University Hospital (TUH) / The Adelaide and Meath Hospital, Dublin, incorporating the National Children’s Hospital (AMNCH), Dublin, Ireland; 5 Vascular Neurology Research Foundation, Tallaght University Hospital (TUH) / The Adelaide and Meath Hospital, Dublin, incorporating the National Children’s Hospital (AMNCH), Dublin, Ireland; 6 Dept. of Radiology, Tallaght University Hospital (TUH) / The Adelaide and Meath Hospital, Dublin, incorporating the National Children’s Hospital (AMNCH), Dublin, Ireland; 7 Dept. of Geriatric and Stroke Medicine, Naas General Hospital, Naas, Ireland; 8 Dept. of Clinical Neurosciences, Royal Free Campus, UCL Queen Square Institute of Neurology, London, UK; 9 Academic Unit of Neurology, School of Medicine, Trinity College Dublin, Dublin, Ireland; 10 Present Address: Vascular Neurology Research Foundation, c/o Department of Neurology, Tallaght University Hospital /AMNCH, Tallaght, Dublin, 24 Ireland

**Keywords:** Carotid endarterectomy and endovascular treatment, Neurovascular multidisciplinary team meeting, Optimal medical therapy, Stroke, TIA

## Abstract

**Background/aims:**

Data are limited on the frequency of ‘consensus decisions’ between sub-specialists attending a neurovascular multidisciplinary meeting (MDM) regarding management of patients with extracranial carotid/vertebral stenoses and post-MDM ‘adherence’ to such advice.

**Methods:**

This prospective audit/quality improvement project collated prospectively-recorded data from a weekly Neurovascular/Stroke Centre MDM documenting the proportion of extracranial carotid/vertebral stenosis patients in whom ‘consensus management decisions’ were reached by neurologists, vascular surgeons, stroke physicians-geriatricians and neuroradiologists. Adherence to MDM advice was analysed in asymptomatic carotid stenosis (ACS), symptomatic carotid stenosis (SCS), ‘indeterminate symptomatic status stenosis’ (ISS) and vertebral artery stenosis (VAS) patients, including intervals between index event to MDM + / − intervention.

**Results:**

One hundred fifteen patients were discussed: 108 with carotid stenosis and 7 with VAS. Consensus regarding management was noted in 96.5% (111/115): 100% with ACS and VAS, 96.2% with SCS and 92.9% with ISS. Adherence to MDM management advice was 96.4% (107/111): 100% in ACS, ISS and VAS patients; 92% (46/50) in SCS patients. The median interval from index symptoms to revascularisation in 50–99% SCS patients was 12.5 days (IQR: 9–18.3 days; *N* = 26), with a median interval from MDM to revascularisation of 5.5 days (IQR: 1–7 days). Thirty patients underwent revascularisation. Two out of twenty-nine patients (6.9%) with either SCS or ISS had a peri-procedural ipsilateral ischaemic stroke, with no further strokes/deaths during 3-months follow-up.

**Conclusions:**

The high frequency of inter-specialty consensus regarding management and adherence to proposed treatment supports a collaborative/multidisciplinary model of care in patients with extracranial arterial stenoses. Service development should aim to shorten times between MDM discussion-intervention and optimise prevention of stroke/death.

## Introduction

National and international guidelines recommend that decisions regarding surgical or endovascular intervention in patients with extracranial carotid or vertebral artery stenosis should be made by a multidisciplinary team (MDT), preferably including a neurologist or stroke physician, vascular surgeon and a neuroradiologist [1, 2]. It has also been recommended that suitable patients with ≥ 50% symptomatic carotid artery stenosis (SCS) be considered for revascularisation within 2 weeks of symptom onset [2–5], with careful selection of higher risk patients with ≥ 60% asymptomatic carotid stenosis (ACS) who may warrant revascularisation [2, 5]. Expert consensus advice from the ESO 2021 guidelines on endarterectomy and stenting for carotid artery stenosis suggests that the independently-assessed **in-hospital** risk of peri-operative stroke or death following endarterectomy for SCS patients should ideally be ≤ 4%, with corresponding risks of ≤ 2% in ACS patients [5]. The ESVS 2017 and ESVS 2023 guidelines recommend that the **30-day** peri-procedural risk of stroke or death in patients with 50–99% SCS who undergo revascularisation should be ≤ 6%, with corresponding risks of ≤ 3% in patients with ≥ 60% ACS [2], [6]. Neurovascular multidisciplinary team meetings (MDMs) have the potential to improve the selection of individual asymptomatic and symptomatic patients who are best suited to optimal medical or interventional treatment, referral for inclusion in ongoing research studies/trials, or a combination of these options to optimise protection against transient ischaemic attack (TIA) or stroke [7–10]. However, some surgeons or interventional neuroradiologists without an established MDM at their hospital might be concerned that discussion of their patients at such meetings might actually reduce the number of patients selected for revascularisation. These concerns may be understandably fuelled by the paucity of available data on the frequency of consensus opinion amongst different subspecialists at MDMs, on the adherence to the advice offered and on clinical outcomes following discussion at such meetings.

## Aims

The aims of this multi-centre audit and quality assessment and improvement process were the following:

- 1. Assess the proportion of patients in whom a consensus management decision was reached by the attending surgeons and physicians from different specialities.
- 2. Assess the ‘adherence’ to the advice offered regarding treatment by the attending physician/surgeon after they had further discussions with the patient following the MDM.
- 3. Document the categories of medical or interventional advice provided to patients with extracranial carotid or vertebral artery stenosis at a neurovascular MDM.
- 4. Assess the time from symptom onset to MDM discussion and the time from MDM discussion to intervention, as appropriate.
- 5. Assess short- and medium-term outcomes in our cohort following MDM discussion.

## Hypotheses

We hypothesised the following:

- 1. Consensus decisions regarding management would be reached in the majority of patients.
- 2. Most treating physicians/surgeons and patients would ‘adhere’ to the primary treatment advice offered at the neurovascular MDM.
- 3. The majority of SCS patients would have revascularisation and the majority of ACS and vertebral artery stenosis (VAS) patients would have optimal medical treatment as their primary recommended treatment.
- 4. Our practice would be in keeping the international guidelines regarding optimal time from symptom onset and MDM discussion + / − intervention, where necessary.
- 5. Outcome event rates before discharge and during medium-term follow-up would be in keeping with the recommended acceptable thresholds suggested by international guidelines.
- We planned to compare our practice and outcomes against recommendations of the ESO 2021 guidelines [5] and the ESVS 2017 and 2023 guidelines [2, 6].

## Methodology

Data from a well-established, weekly neurovascular MDM at our university teaching hospital, a secondary and tertiary regional referral centre for neurovascular patients, were prospectively collected between 21st of Sept 2017 and 27th of Feb 2020 (prior to the initial COVID-19 pandemic in Ireland) for this audit and quality improvement (QI) project. The meeting is run in close collaboration with our attending consultant neurologists, consultant vascular surgeons, consultants in older adult/stroke medicine and consultant diagnostic neuroradiologists. At this meeting, the attending consultants have the opportunity to discuss any patients with extracranial carotid and/or vertebral artery stenosis who may be suitable for revascularisation with endarterectomy or endovascular treatment, or in whom they feel optimal medical therapy may be the best treatment option. If very urgent treatment decisions regarding endarterectomy or endovascular treatment need to be made in clinically unstable patients prior to the weekly neurovascular MDM, the guideline at our hospital is that such cases must be discussed by a consultant vascular surgeon with either a consultant neurologist or stroke physician to reach consensus between the two consultants in question before proceeding to intervention. This audit/quality assessment and improvement process were coordinated by the chair of the neurovascular MDM (an experienced vascular neurologist with a subspecialty interest in carotid and vertebral artery stenosis; DJHM) and a consultant vascular surgeon (with extensive expertise in managing patients with carotid, vertebral and other extracranial stenoses; ST).

The data were entered prospectively onto a standardised MDM proforma, which included a summary of the clinical history, known prescribed medications and key examination findings, neurovascular investigations, the treating clinician’s final decision regarding the symptomatic status of the artery(ies) in question, ABCD^2^ scores in TIA patients (range: 0–7) and modified Rankin scale scores in living patients who had experienced an ischaemic stroke (range: 0–5).

For the purpose of this study, patients were included in the ACS group if they were incidentally noted to have extracranial internal carotid stenosis on vascular imaging which had not caused any relevant neurovascular presenting symptoms prior to MDM discussion; the ACS was typically noted on colour Doppler ultrasound and confirmed on computed tomography angiography (CTA) or magnetic resonance angiography (MRA) in the majority of cases. Patients were included in the symptomatic carotid stenosis group if they had experienced a TIA or ischaemic stroke in the vascular territory supplied by an ipsilateral carotid artery stenosis of any severity which prompted MDM discussion, with no other cause for their symptoms identified. Patients were defined as having carotid stenosis of ‘indeterminate symptomatic status (ISS)’ if it was unclear on clinical grounds whether the ipsilateral carotid artery (ICA) stenosis had caused their neurovascular symptoms or if they had more than one potential cause for their TIA/stroke in the vascular territory supplied by an ICA stenosis (e.g. ICA stenosis and atrial fibrillation). Patients were defined as having VAS if they had a TIA or ischaemic stroke in the vascular territory supplied by a stenosed extracranial vertebral artery on either side.

The severity of arterial stenosis using extracranial computed tomography angiography (CTA) or magnetic resonance angiography (MRA) was formally quantified on the day of the MDM by a neuroradiologist (or by the chairing vascular neurologist in his/her absence (DJHM)) according to the North American Symptomatic Carotid Endarterectomy Trial (NASCET) method of estimating stenosis [11]. Stenoses were subsequently grouped into categories as being either ‘normal’ (< 30%), ‘mild’ (≥ 30–49%), ‘moderate’ (≥ 50–69%), ‘severe’ (≥ 70–99%) or occlusion (100%). All available data on assessment of carotid and vertebral stenoses with colour Doppler ultrasound of neck arteries were also prospectively recorded at the MDM, with categorisation of stenosis equating with assessment of stenosis severity using the NASCET criteria [12]. Findings on intracranial CTA, MRA or formal catheter angiography, where available, and whether or not patients had evidence of carotid or vertebrobasilar territory ischaemia or infarction on CT and/or MRI brain were also prospectively recorded. Overall management advice and whether a consensus management decision was reached by all Consultants attending the MDM were documented. The categories of advice included: carotid endarterectomy (CEA), endovascular treatment (EVT), optimal medical therapy (OMT), change medical therapy (CMT), further investigations (further Ix), re-discussion and ‘Non-Consensus’. A ‘consensus management decision’ was defined as complete agreement between all attending consultants regarding treatment with revascularisation or OMT alone. ‘Adherence’ was defined as following through with the consensus advice provided at the MDM by the attending consultant regarding revascularisation or OMT after further discussion with the patient for management of ACS, SCS, ISS or VAS.

It was the practice at our centre during the audit period that all patients were assessed by an attending neurologist or stroke physician before and after any revascularisation procedure had been performed by our vascular surgeons to independently identify any peri-procedural outcomes; relevant findings were recorded in the hospital notes. We collated all available contemporaneously recorded data from the hospital notes and/or electronic case records on our server to assess the frequency of the ‘**primary composite clinical outcome**’ of peri-procedural/in-hospital stroke or death before discharge, as well as other pre-specified peri-procedural complications documented by the treating physician/surgeon. These included recurrent ipsilateral/contralateral/territorial TIA; and recurrent ipsilateral/contralateral/territorial ischaemic or haemorrhagic stroke. We also analysed available data on pre-specified ‘**secondary clinical outcomes**’ which had been recorded by the clinical teams during routine clinical follow-up, and which were either listed on the MDM proforma, and/or recorded in the hospital notes or electronic case records: stroke or death alone during follow-up between discharge and the 3-month follow-up visit; transient ischaemic attack, stroke or death between discharge and the 3 month follow-up visit in SCS patients, ISS and ACS patients; recurrent ipsilateral or contralateral territorial TIA; confirmed MI/coronary revascularisation (stenting/CABG); vascular death/other causes of death; consultant-confirmed disabling cranial nerve palsy/nerve injury; significant access-site haematoma; confirmed hyperperfusion syndrome; or other causes of death between discharge and the 3-month follow-up visit. If patients were only followed up at a referring centre after discharge, their attending consultant stroke physician was contacted to enquire about the occurrence of any of the above outcome events after the MDM.

The time intervals from symptom onset to MDM discussion, and from MDM discussion to intervention in appropriate cases, were calculated to determine whether we were treating patients in line with best international practice according to recent clinical practice guidelines [13]. All data were entered onto a centralised excel spreadsheet prior to final analysis. The percentage of patients with ≥ 50–99% SCS who experienced (i) a peri-procedural primary clinical outcome in-hospital and (ii) secondary clinical outcomes during follow-up for 3 months was compared with the recommended thresholds laid out in the ESVS 2017, ESVS 2023 and ESO 2021 clinical practice guidelines to assess our centre’s performance against international best practice. We also prospectively planned to assess the percentages of patients with ≥ 50–99% ACS who experienced the above clinical outcomes to compare their risk of outcomes with the recommended thresholds laid out in the ESVS 2017, ESVS 2023 and ESO 2021 clinical practice guidelines.

## Ethical approval/clinical audit committee registration

Because these data were collated as part of a prospective audit and quality assessment and improvement programme, our Local Research Ethics Committee (LREC) hospital guidelines indicate that formal LREC approval for such a study is not required. However, the study was registered with the Tallaght University Hospital-AMNCH clinical audit committee, all data were securely stored in electronic format, and all presented data were fully pseudonymised so that no individual patient could be identified from the data contained within this manuscript. The study and analyses were conducted in accordance with best ethical practice and supervised by an experienced consultant who is International Conference on Harmonisation of Technical Requirements for Registration of Pharmaceuticals for Human Use Good Clinical Practice (ICH GCP)-certified.

## Statistical methodology

Paired or unpaired *t* tests were used for comparison of paired and unpaired parametric variables, respectively. The Wilcoxon signed rank test and the Wilcoxon rank sum test were used for comparison of paired and unpaired non-parametric variables, and the Kruskal–Wallis rank sum test was used for comparison of multiple non-parametric variables, where appropriate. Chi-squared or Fisher exact tests were used to compare proportions between groups, where appropriate. All statistical calculations were performed using SPSS^®^ (SPSS Version 27, 2020) and PRISM 9 software (GraphPad Software Inc).

## Results

One hundred and fifteen patients were discussed at our weekly neurovascular MDM over a 29-month period (21/09/2017–27/02/2020): 108 with carotid stenosis and 7 with VAS. Demographic data are displayed in Table 1. Overall, a consensus decision regarding management was reached in 111/115 cases (96.5%): 28/28 (100%) patients with ACS, 50/52 (96.2%) with SCS, 26/28 (92.9%) with ISS and 7/7 patients (100%) with VAS (Table 2). Non-consensus regarding management was seen in 4 (3.5%) cases in the SCS and ISS groups combined (Table 2).

**Table 1:** Demographic and vascular risk profiles of study participants at the time of the Neurovascular MDM

**Parameter**	**CVD patients** **(*N* = 115)**
Mean age in years	70.24
Sex (M/F)	84/31
Aspirin	84/114 (74%)
Clopidogrel	15 (13%)
Aspirin & dipyridamole	32 (28%)
Aspirin & clopidogrel	12 (10%)
Prior TIA	25 (21.7%)
Prior stroke	19 (16.5%)
IHD*	29/114 (25.4%)
Hypertension	81/111 (72.9%)
Diabetes mellitus	33/114 (28.9%)
Hyperlipidaemia**	81/107 (75.7%)
Atrial fibrillation/flutter	19 (16.5%)
Prior DVT/PE***	5 (4.3%)
Peripheral vascular disease	6 (5.2%)
Migraine with or without aura	10 (8.6%)
Current smoker	29/94 (30.8%)
Ex-smoker	36/94 (38.2%)
Never smoker	26/94 (27.6%)
Antiphospholipid syndrome	2 (1.7%)
Bleeding diathesis	0
Rheumatic fever	1 (0.8%)

Values are means (+ / − SD) or absolute values with percentages in parentheses (%), where appropriate

*CVD* cerebrovascular disease, IHD* history of ischaemic heart disease, Hyperlipidaemia** total cholesterol > 5.0 mmol/L or LDL > 3.5 mmol/L at the time of the study design, DVT/PE*** deep venous thrombosis/pulmonary embolism. Vascular risk factor data were not available for presentation at the time of the MDM in some patients, so the denominator is specified if it is < 115 in some rows

**Table 2:** Categories of advice provided and the frequency of consensus management decisions in patients with carotid and vertebral artery stenosis (*N* = 115)

**Parameter**	**All** ***N* = 115 (%)**	**ACS** ***N* = 28 (%)**	**SCS** ***N* = 52 (%)**	**ISS** ***N* = 28 (%)**	**VAS** ***N* = 7 (%)**
**CEA/ EVT**	**28 (24.3)**	**0**	**CEA 26 (50)** **EVT 1 (1.9)**	**1 (3.6)**	**0**
			< 50%: 0	< 50%: 0	
			≥ 50–69%: 5	≥ 50–69%: 0	
			≥ 70–99%: 22	≥ 70–99%: 1	
			100%: 0	100%: 0	
**OMT**	**75 (65.2)**	**27 (96.4)**	**20 (38.5)**	**21 (75)**	**7 (100)**
		< 50%: 4	< 50%: 3	< 50%: 5	< 50%: 0
		≥ 50–69%: 7	≥ 50–69%: 3	≥ 50–69%: 9	≥ 50–69%: 1
		≥ 70–99%: 13	≥ 70–99%: 5	≥ 70–99%: 4	≥ 70–99%: 5
		100%: 0	100%: 7	100%: 2	100%: 1
		N/A^#^: 3	N/A^#^: 2	N/A^#^: 1	
**Further Ix**	**8 (7)**	**1 (3.6)**	**3 (5.8)**	**4 (14.3)**	**0**
		< 50%: 0	< 50%: 0	< 50%: 0	
		≥ 50–69%: 0	≥ 50–69%: 2	≥ 50–69%: 0	
		≥ 70–99%: 1	≥ 70–99%: 1	≥ 70–99%: 3	
		100%: 0	100%: 0	100%: 0	
				N/A^#^: 1	
**Rediscussion**	**4 (3.5)**	**0**	**2 (3.8)**	**2 (7.1)**	**0**
			< 50%: 0	< 50%: 0	
			≥ 50–69%: 0	≥ 50–69%: 1	
			≥ 70–99%: 2	≥ 70–99%: 1	
			100%: 0	100%: 0	
**Consensus**	**111 (96.5)**	**28 (100)**	**50 (96.2)**	**26 (92.9)**	**7 (100)**
		< 50%: 5	< 50%: 4	< 50%: 5	< 50%: 2
		≥ 50–69%: 7	≥ 50–69%: 8	≥ 50–69%: 9	≥ 50–69%: 3
		≥ 70–99%: 14	≥ 70–99%: 31	≥ 70–99%: 8	≥ 70–99%: 1
		100%: 0	100%: 5	100%: 2	100%: 1
		N/A^#^: 2	N/A^#^: 2	N/A^#^: 2	
**Non-consensus**	**4 (3.5%)**	**0**	**2 (3.8)**	**2 (7.1)**	**0**
			< 50%: 0	< 50%: 0	
			≥ 50–69%: 1	≥ 50–69%: 1	
			≥ 70–99%: 1	≥ 70–99%: 1	
			100%: 0	100%: 0	

Values are absolute numbers (%). The overall numbers in each patient group are presented at the top of the cell in each column, followed by data which are subcategorised accordingly to the severity of the stenosis of the artery in question

In this and other tables, the following abbreviations apply: *ACS* asymptomatic carotid stenosis, *SCS* symptomatic carotid stenosis, *ISS* indeterminate symptomatic status (carotid) stenosis, *VAS* vertebral artery stenosis, *CEA* carotid endarterectomy, *EVT* endovascular treatment, *OMT* optimal medical therapy, *Ix* investigations

*N/A*^*#*^ not available for this study because CTA or MRA were not done, and we only had CDUS data available at the MDM

Full adherence to advice provided at the MDM was observed in 92% (46/50) of SCS patients, and in all with ACS, ISS and VAS. Three patients in whom revascularisation was recommended did not proceed to intervention and were managed by OMT; one patient who was advised to have OMT was rediscussed and then had further investigations, but did not subsequently undergo urgent revascularisation because the treating vascular surgeon did not feel it was safe to proceed (Table 3).

**Table 3:** Adherence to advice given for carotid and vertebral artery stenosis patients in whom a consensus decision re management was reached (total *N* = 111)

**Parameter**	**All** ***N* (%)**	**ACS** ***N* = 28 (%)**	**SCS** ***N* = 50 (%)**	**ISS** ***N* = 26 (%)**	**VAS** ***N* = 7 (%)**
**CEA or EVT**	25/28 (89.3)	0	CEA 23/26 (88.5) EVT 1/1 (100) Total: 24/27 (88.9)	1/1 (100)	0
**OMT**	75/76 (98.7)	27/27 (100)	20/21 (95.2)	21/21 (100)	7/7 (100)
**Further Ix**	7/7 (100)	1/1 (100)	2/2 (100)	4/4 (100)	0
**Adherence**	107/111 (96.4)	28/28 (100)	46/50 (92)	26/26 (100)	7/7 (100)
**Non-adherence**	4 (3.6)	0	4	0	0
**Non-consensus**	4	0	2	2	0

Revascularisation was recommended in 27/52 (51.9%) patients with SCS, with OMT in 20 (38.5%), and further investigations or rediscussion recommended in 5 (9.6%) of these patients. Revascularisation was only initially recommended in 1/28 (3.6%) patients with ISS who had a ≥ 70–99% ICA stenosis, but in no patients initially with ACS or VAS (Table 2). In the 111 patients in whom a consensus decision was reached at the MDM, 96.4% overall adhered to that advice following the MDM when their treating physician had subsequently discussed matters with them.

Thirty patients had revascularisation; 28 patients underwent revascularisation following a consensus management decision at the MDM, and the remaining 2 patients were subsequently advised to have revascularisation after the MDM. The median interval from symptom onset to MDM discussion in patients with 50–99% SCS who subsequently underwent revascularisation was 9 days (IQR: 7–12.75). The median interval from index symptoms to intervention in patients with 50–99% SCS was 12.5 days (IQR: 9–18.3 days; *N* = 26), with a median interval from MDM discussion to intervention in these SCS patients of 5.5 days (IQR: 1–7 days; Table 4). For patients with 50–99% ISS stenosis, the median interval from index symptoms to intervention was 16 days (IQR: 9.5–18.5 days; *N* = 3), and from MDM to intervention was 1 day (IQR: 1–4.5 days). One patient with a symptomatic left internal carotid artery (LICA) occlusion and minor ischaemic stroke, who also had 80% right internal carotid artery (RICA) ACS, subsequently underwent deferred right CEA 2.5 months after MDM re-discussion, completion of further investigations and after recovery from a LICA territory stroke, so the time interval from MDM to intervention was 75 days.

**Table 4:** Median interval in days (interquartile range (IQR)) from (a) index TIA/ischaemic stroke symptoms to revascularisation and (b) MDM discussion to revascularisation in patients with 50–99% stenosis (*N* = 30 because 2 additional patients proceeded to revascularisation after the MDM)

**Parameter**	**ACS** ***N* = 1**	**SCS** ***N* = 26**	**ISS** ***N* = 3**
**Index TIA/stroke to** **revascularisation** **(days [IQR])**	N/A	12.5 [9–18.3]	16 [9.5–18.5]
**MDM to** **revascularisation** **(days [IQR])**	75	5.5 [1–7]	1 [1–4.5]

Two of 26 SCS patients (7.6%) who underwent revascularisation, or two of 29 patients with either SCS or ISS (6.9%) who underwent revascularisation had a peri-procedural primary clinical outcome of ipsilateral ischaemic stroke (Table 5). The first of these two SCS patients with moderate SCS experienced a non-disabling post-operative ischaemic stroke; MRS score increased from 1 pre-operatively to 2 post-operatively. He had been on aspirin + clopidogrel combination therapy at the time of MDM, but clopidogrel was held for 7 days pre-operatively and CEA performed on aspirin monotherapy based on the decision by the treating consultant. The second patient with severe SCS had a disabling intra-operative stroke whilst on aspirin + dipyridamole combination therapy throughout the peri-operative period; MRS was 0 pre-operatively, not documented post-operatively, but post-operative NIHSS score was 14 (Table 5). Only 1/26 patients (3.8%) with severe SCS had a secondary clinical outcome before discharge: a consultant-confirmed disabling hypoglossal nerve palsy and access-site hematoma noted on the day of the CEA (Table 5).

**Table 5:** Peri-procedural in-hospital primary outcome (stroke or death) and secondary clinical outcomes (confirmed MI/coronary revascularisation (stenting/CABG); vascular death/other causes of death; consultant-confirmed disabling cranial nerve palsy/nerve injury; significant access-site haematoma; confirmed hyperperfusion syndrome; or other causes of death) in SCS or ISS patients who underwent revascularisation and their respective % stenosis categories on extracranial CTA (*N* = 26 SCS and *N* = 3 ISS patients)

	**SCS (*N* = 26)** **number (%)**	**% Stenosis**	**ISS (*N* = 3)** **number (%)**	**% Stenosis**
**Peri-** **procedural** **primary** **outcome**	2 (7.6%)	≥ 50–69%: 1^a^	0 (0%)	≥ 50–69%: 0
		≥ 70–99%: 1^b^	0 (0%)	≥ 70–99%: 0
**Peri-** **procedural** **secondary** **outcome**	1 (3.8%)	≥ 70–99%: 1^c^	0 (0%)	≥ 70–99%: 0

Of note, none of the patients who underwent revascularisation died during the peri-procedural period in hospital

^a^1 ipsilateral ICA territory ischaemic stroke (MRS pre-operatively = 1; MRS post-operatively = 2)

^b^1 ipsilateral ICA territory ischaemic stroke (MRS pre-op = 0; NIHSS post-op = 14)

^c^1 consultant-confirmed cranial nerve (hypoglossal) palsy and access-site hematoma

Two of twenty-six SCS patients (7.6%) had complex presentations with their stroke, one of whom had haemorrhagic transformation of a large ipsilateral infarct resulting in a delay to revascularisation with CEA until 61 days after index symptom onset (interval of 7 days between MDM discussion and successful CEA). The other patient with LICA SCS presented with a left MCA territory ischaemic stroke with right sided weakness and sensory inattention. The patient was initially treated with aspirin monotherapy by his treating physician, but deteriorated overnight with embolisation to the ipsilateral left MCA causing aphasia prompting transfer to the National Thrombectomy Centre for urgent MCA mechanical thrombectomy and LICA stenting the following day without clinical improvement. Because no initial haemorrhagic transformation was noted on repeat imaging, clopidogrel was added to aspirin therapy. This patient, who was the only one in this series treated with urgent stenting, subsequently developed delayed symptomatic haemorrhagic transformation of a left hemispheric infarct on aspirin + clopidogrel combination therapy and prophylactic subcutaneous enoxaparin 17 days after admission, requiring rationalisation of antithrombotic therapy.

One hundred and two of 115 patients (88.7%) with carotid or vertebral artery stenosis had complete 3-month follow-up data available (Table 6). There were no strokes or deaths between discharge and the 3-month follow-up visit. However, 3/102 patients (2.9%) had delayed secondary clinical outcomes between discharge/MDM discussion and their 3-month follow-up visit. Two patients had subsequent TIAs. One ACS patient had a LICA territory haemodynamic TIA distal to a longstanding LICA occlusion and contralateral to a moderate post-stenting RICA restenosis; one patient with mild RICA SCS had recurrent left monocular TIAs in the contralateral left ICA territory, felt to be cardioembolic in association with rheumatic mitral valve disease (Table 6). Therefore, 1 of 22 (4.5%) ACS patients and 1 of 51 SCS patients (1.9%) who had available follow-up data had recurrent **contralateral cerebrovascular events** during our follow-up period, but all were treated with modifications of their medical therapy regimens, and none underwent revascularisation after assessment by their treating physicians or surgeons. One of 24 ISS patients (4.2%) in whom follow-up data were available had a confirmed ST-elevation myocardial infarction requiring coronary stenting and a change in his antiplatelet therapy from aspirin + dipyridamole MR to aspirin + clopidogrel combination therapy.

**Table 6:** Clinical outcomes between discharge from hospital and the 3-month follow-up visit (*N* = 102 patients)

**Parameter**	**All** ***N* = 102 (%)**	**ACS** ***N* = 22 (%)**	**SCS** ***N* = 51 (%)**	**ISS** ***N* = 24 (%)**	**VAS** ***N* = 5 (%)**
**Stroke/death at 3 months**	0	0	0	0	0
**3-month secondary outcomes of ipsilateral ICA territory TIA**	0	0	0	0	0
**3-month secondary outcomes of TIAs outside of the ipsilateral ICA territory**	2 (1.9)	1 (4.5)	1 (1.9)	0	0
		< 50%: 0	< 50%: 1		
		≥ 50–69%: 1	≥ 50–69%: 0		
		≥ 70–99%: 0	≥ 70–99%: 0		
		100%: 0	100%: 0		
**Other 3-month secondary outcomes: ST-elevation myocardial infarction requiring percutaneous coronary intervention and stenting (PCI)**	1 (0.9)	0	0	1 (4.2)	0
				< 50%: 0	
				≥ 50–69%: 1	
				≥ 70–99%: 0	
				100%: 0	
**No outcome events**	99 (97.1)	21 (95.5)	50 (98)	23 (95.8)	5 (100)

## Discussion

This prospective audit/QI project has shown the important finding that a neurovascular MDM can enable colleagues in clinical practice in neurology/stroke medicine, vascular surgery and neuroradiology to openly discuss and reach a consensus decision regarding evidence-based management in the vast majority (96.5%) of patients with extracranial carotid or vertebral stenoses. This should encourage the establishment of a neurovascular MDM at Neurovascular/Stroke Centres where such a service does not exist, as advised in recent clinical practice guidelines [2, 5, 6], with the attendant opportunities for closer collaboration, audit based on independent assessment of outcomes, and research in this field to enhance patient care.

Furthermore, most treating physicians/surgeons (96.4%) adhered to the main treatment advice offered at the neurovascular MDM. This is similar to the level of adherence seen in SCS patients (94%), but higher than the level of adherence observed in ACS patients (69%) in a prior study by Rimmele et al. [13]. Our higher level of adherence in ACS patients undoubtedly also reflects the fact that we were not participating in randomised controlled trials (RCTs) of revascularisation vs. OMT in patients with ACS or VAS during the audit/QI study period, so our findings were not influenced by patients potentially declining participation in RCTs after the MDM. It is also probable that there were other unspecified differences in practice regarding the extent of investigations which were performed **before** initial discussion at the MDM in the two studies, because we less frequently recommended doing further investigations in ACS or SCS patients than Rimmele et al.

Revascularisation was recommended in the majority of SCS patients at our neurovascular MDM (51.9%), in keeping with international guidelines [2, 5, 6] and the main findings of a retrospective audit of a neurovascular MDM in SCS patients in Germany [13]. We had anticipated recommending revascularisation in an even higher proportion of patients with SCS overall, but this was not felt to be safe or feasible due to other co-morbidities in several patients whom we encountered in routine clinical practice, including patients with carotid near occlusion and those with moderate carotid stenosis. During follow-up, only 1/51 (1.9%) of the SCS patients on OMT had a contralateral TIA, and this patient did not warrant revascularisation. However, these data illustrate that this subgroup of patients have a clinically important risk of having recurrent cerebrovascular events on modern medical treatment and warrant ongoing clinical monitoring by their treating physicians/surgeons to fully investigate the precise cause of any recurrent cerebrovascular events and to optimise secondary prevention over time.

In contrast, all patients with ACS and VAS were initially advised to have OMT following discussion at our MDM. Only one of 22 ACS patients (4.5%) with moderate restenosis had a recurrent haemodynamic TIA during follow-up which the attending physician opted to treat with alteration of optimal medical therapy. Therefore, the proportion of ACS patients in whom revascularisation was ultimately recommended was much lower at our centre (0% at the MDM pending further investigations and 4.5% (1/22) after completion of investigations) than at a university medical centre in Germany (26.8% of cases) [13]. Aside from the prospective nature of our data collection and the general tendency of clinicians at our centre to treat most ACS patients with OMT, the disparity between our findings and the recommendations of that previously published audit [13] is also partly explained by the fact that our centre was not randomising ACS patients to the SPACE-2 [13, 14], ACST-2 [15] or CABACS [16] trials during this time period. The absence of any cerebrovascular events in our ACS subgroup over 3 months had been anticipated from most [17–21] but not all studies [22].

It is of interest that 75% of patients with ISS who had potential competing mechanisms responsible for their presenting symptoms, and whose management was discussed in this ‘real-world’ clinical setting at our Neurovascular centre, were also advised to have OMT by the various specialists in attendance (Table 2). This subcategory of ISS patients was not included in the aforementioned retrospective audit [13], and all such patients, e.g. with concomitant atrial fibrillation and carotid stenosis, or symptoms which could not be confidently attributed to an extracranial carotid stenosis were excluded from the major RCTs comparing revascularisation with OMT in patients with carotid stenosis. However, a numerically higher proportion of patients with ISS (21.4%) compared with SCS (9.6%) was advised to undergo further investigations and/or have their case rediscussed by members of our MDM to try to further clarify the most likely aetiology of their TIA/stroke and optimise protection against recurrent vascular events (Table 2). Further multi-centre studies to collate data on management decisions and clinical outcomes in cohorts of ISS patients are warranted to inform future clinical practice because 1/24 (4.2%) of these patients also had a TIA during the 3-month follow-up period.

Our recommendation to revascularise the majority of SCS patients with CEA rather than with EVT is in keeping with recent clinical practice guidelines [2, 5, 6] and is supported by data from randomised controlled trials of CEA vs. EVT in SCS patients with 50–99% stenosis [23, 24].

Between 2005 and 2013, many vascular surgeons transitioned to performing most CEAs within 2 weeks of symptom onset, with the mean time to surgery decreasing from 25 to 6 days [25]. The median interval from index symptoms to revascularisation in our 50–99% SCS patients of 12.5 days is in keeping with recent international clinical practice guidelines which recommended that CEA be performed preferably within 2 weeks of symptom onset [2–6, 25–28]. The median interval from MDM to revascularisation in patients with 50–99% SCS was 5.5 days, partly due to the fact that our vascular surgeons do not have routine access to ‘protected emergency theatre slots’ for patients warranting urgent carotid endarterectomy outside of their scheduled vascular surgery lists. Importantly, one SCS patient had a large infarct with haemorrhagic transformation which necessitated empirically delaying revascularisation to limit the risk of further haemorrhagic transformation and hyperperfusion injury [3], thus emphasising the fact that it is not always appropriate to intervene on all SCS patients within 2 weeks of symptom onset [2, 3]. This median interval to intervention is longer than the interval of 3 days from initial assessment by a consultant stroke physician in a TIA clinic to urgent CEA by a vascular surgeon in one large UK single centre audit [29]. However, the datasets are not directly comparable because, to our knowledge, although patients in that study in the UK were clearly treated in a collaborative, multidisciplinary manner by their expert clinicians, they were not discussed at a dedicated neurovascular MDM *per se*.

The observed risk of peri-operative in-hospital stroke following CEA was 7.6% in 50–99% SCS patients and 6.9% in the SCS and ISS subgroups combined, with no deaths. This risk is higher than the recently-proposed ‘recommended threshold’ of < 4% for the independently-assessed risk of in-hospital stroke/death [5, 30], and only slightly higher than the overall 30-day recommended threshold of < 6% for stroke/death in patients with 50–99% SCS following CEA or EVT [2, 6], because no revascularised SCS patients had a recurrent stroke between discharge and their 3-month follow-up visit (Table 5). In one study which collated data from 2 prospective audit periods from predominantly symptomatic patients who underwent a CEA in Scottish NHS hospitals, in which 89% of patients had ≥ 70% stenosis overall, the combined in-hospital risk of stroke or death appeared to be 4.3% [31]. However, these figures were derived from outcome data reported by treating vascular surgeons alone which were not verified by an independent assessor; the authors acknowledged that this might have led to under-estimation of outcome events in their series. In a Dutch National Audit for Carotid Interventions, the mean risk of any stroke or death in hospital or within 30 days of CEA for ‘high-grade carotid artery stenosis’ was 3.6%, but the ‘casemix-adjusted’ risk of this outcome of stroke or death varied between 0 and 9.4% between participating hospitals in the Netherlands [32]. Therefore, the risk of in-hospital stroke or death of 6.9% observed in this study in patients with 50–99% SCS or ISS who underwent revascularisation at our centre falls within the overall range observed in the Netherlands. All patients in our study were independently assessed following surgery by their treating neurologist/stroke physician, which has previously been shown to increase the risk of recorded peri-operative outcomes compared with assessment by a vascular surgeon alone [6, 33]. Nevertheless, these data will be used by our group to potentially identify and minimise any risk factors which could contribute to a future risk of peri-procedural stroke [34].

This prospective audit and QI project had some **potential limitations**. Neurological outcomes either in the peri-procedural period in patients undergoing revascularisation, or during follow-up in those who were treated with revascularisation or OMT were recorded by the primary treating neurologist/stroke physician and were not verified further by another ‘independent’ neurologist/stroke physician who was not involved in the patients’ care; limited subspecialty staffing on site and in our region precluded such an approach. However, based on the aforementioned practice of independent assessment of all patients with carotid stenosis before and after revascularisation by the attending neurologist/stroke physician who collaborated with the attending vascular surgeon to deliver care, we believe that this audit reflects the findings of a real-world practice with independent objective assessment of outcome measures [30, 33]. Furthermore, although all of the data from the MDM itself were recorded prospectively on the day of the meeting, designated rotating vascular neurology residents in training participated in collecting the 3-month outcome data for this audit by reviewing available electronic letters on our clinical server or the hospital notes, as required. Three-month follow-up data were not available in 11.3% of patients (13/115), all of whom were treated with OMT, so it is possible that we might have underestimated outcomes in this subgroup of patients. In a selected number of patients who were not followed up at our main Neurovascular/Stroke Centre, the residents and the supervising author contacted the external collaborating primary referring physicians to request any missing data. This could have created the potential for recall bias because the referring physicians had to check their own hospital records or check with some patients whether they had experienced 3-month outcome events during follow-up. Nevertheless, these outcome assessments were still performed independent of the treating vascular surgeon, so this is ultimately considered to be a strength of this study. We have relatively limited data on patients with vertebral artery stenoses, likely reflective of selection bias by referring physicians who mainly focused on discussing management of patients with carotid stenosis at this MDM. Because data collection was mainly performed at a single Neurovascular/Stroke Centre which accepted referrals from other hospitals in the region, further studies are clearly warranted to clarify whether collation of supra-regional or national data would produce similar or different results to further guide practice in this field. National audits of data specifically from patients with carotid artery stenosis who should be discussed at a neurovascular MDM, with outcomes assessed by a neurologist/expert stroke physician independent of the treating vascular surgeon/interventionist, have the potential to drive improvements in clinical diagnosis, management and response to treatment. Such data would also enable comparisons of outcomes between healthcare providers, within and between countries, identification of areas for quality improvement in certain centres, and should encourage adherence to international evidence-based management guidelines [32]. Finally, these data were collected before the onset of the COVID-19 pandemic, so the impact of COVID-19 on the volume of referrals for MDM discussion, consensus management decisions, advice provided and adherence to such advice warrants assessment.

## Conclusions

The high frequency of inter-specialty consensus regarding management and adherence to proposed treatment supports a collaborative, multidisciplinary model of care in patients with extracranial arterial stenoses. Future service development should aim to shorten the time interval between MDM discussion and revascularisation, where appropriate, to optimise primary and secondary prevention of TIA or stroke, and to minimise peri-procedural risks of stroke or death during carotid endarterectomy.

## Data Availability

Original source data from our study are available on request to the first author to be shared with qualified scientific collaborators for relevant research projects.
