# Correction to: Frequency of inter‑specialty consensus decisions and adherence to advice following discussion at a weekly neurovascular multidisciplinary meeting

**DOI:** 10.1007/s11845-023-03396-5

**Published:** 2023-06-03

**Authors:** Chika Offiah, Sean Tierney, Bridget Egan, Ronán D. Collins, Daniel J. Ryan, Allan J. McCarthy, Deirdre R. Smith, James Mahon, Emily Boyle, Holly Delaney, Rory O.’Donohoe, Alison Hurley, Richard A. Walsh, Sinead M. Murphy, Petya Bogdanova‑Mihaylova, Sean O.’Dowd, Mark J. Kelly, Taha Omer, Tara Coughlan, Desmond O’Neill, Mary Martin, Stephen J. X. Murphy, Dominick J. H. McCabe

**Affiliations:** 1 Dept. of Neurology, Tallaght University Hospital (TUH) / The Adelaide and Meath Hospital, Dublin, incorporating the National Children’s Hospital (AMNCH), Dublin, Ireland; 2 Stroke Service, Tallaght University Hospital (TUH) / The Adelaide and Meath Hospital, Dublin, incorporating the National Children’s Hospital (AMNCH), Dublin, Ireland; 3 Dept. of Vascular Surgery, Tallaght University Hospital (TUH) / The Adelaide and Meath Hospital, Dublin, incorporating the National Children’s Hospital (AMNCH), Dublin, Ireland; 4 Dept. of Age‑Related Health Care, Tallaght University Hospital (TUH) / The Adelaide and Meath Hospital, Dublin, incorporating the National Children’s Hospital (AMNCH), Dublin, Ireland; 5 Vascular Neurology Research Foundation, Tallaght University Hospital (TUH) / The Adelaide and Meath Hospital, Dublin, incorporating the National Children’s Hospital (AMNCH), Dublin, Ireland; 6 Dept. of Radiology, Tallaght University Hospital (TUH) / The Adelaide and Meath Hospital, Dublin, incorporating the National Children’s Hospital (AMNCH), Dublin, Ireland; 7 Dept. of Geriatric and Stroke Medicine, Naas General Hospital, Naas, Ireland; 8 Dept. of Clinical Neurosciences, Royal Free Campus, UCL Queen Square Institute of Neurology, London, UK; 9 Academic Unit of Neurology, School of Medicine, Trinity College Dublin, Dublin, Ireland; 10 Present Address: Vascular Neurology Research Foundation, c/o Department of Neurology, Tallaght University Hospital /AMNCH, Tallaght, Dublin 24, Ireland

**Correction to: Irish Journal of Medical Science (2023)** **https://doi.org/10.1007/s11845-023-03319-4**

The original article contains an error during online publication. There are minor and some major errors in the text/tables (tables in particular, several of which are mal-aligned). These errors has been modified and finalized.

The original article has been corrected.

